# Coordination during group departures and progressions in the tolerant multi-level society of wild Guinea baboons (*Papio papio*)

**DOI:** 10.1038/s41598-021-01356-6

**Published:** 2021-11-09

**Authors:** Davide Montanari, William J. O’Hearn, Julien Hambuckers, Julia Fischer, Dietmar Zinner

**Affiliations:** 1 Cognitive Ethology Laboratory, German Primate Center, Kellnerweg 4, 37077 Göttingen, Germany; 2 HEC Liège, University of Liège, Liège, Belgium; 3 Department of Primate Cognition, Georg-August-Universität Göttingen, 37077 Göttingen, Germany; 4 Leibniz ScienceCampus Primate Cognition, 37077 Göttingen, Germany

**Keywords:** Social evolution, Animal behaviour, Behavioural ecology

## Abstract

Collective movement of social groups requires coordination between individuals. When cohesion is imperative, consensus must be reached, and specific individuals may exert disproportionate influence during decision-making. Animals living in multi-level societies, however, often split into consistent social subunits during travel, which may impact group coordination processes. We studied collective movement in the socially tolerant multi-level society of Guinea baboons (*Papio papio*). Using 146 group departures and 100 group progressions from 131 Guinea baboons ranging in Senegal’s Niokolo-Koba National Park, we examined individual success at initiating group departures and position within progressions. Two-thirds of attempted departures were initiated by adult males and one third by adult females. Both sexes were equally successful at initiating departures (> 80% of initiations). During group progressions, bachelor males were predominantly found in front, while reproductively active ‘primary’ males and females were observed with similar frequency across the whole group. The pattern of collective movement in Guinea baboons was more similar to those described for baboons living in uni-level societies than to hamadryas baboons, the only other multi-level baboon species, where males initiate and decide almost all group departures. Social organization alone therefore does not determine which category of individuals influence group coordination.

## Introduction

Many group living animals coordinate the time and direction of their movements to maintain group cohesion^1,2^. Coordination at the group level may arise from individuals following simple rules of self-organization^3–6^. In other instances, social animals coordinate through consensus, where group members collectively choose between mutually exclusive movement options^1,7–11^. When a group member initiates movement in a specific direction, a decision-making situation arises, as other group members may choose whether or not to follow the initiator. If a critical portion of the group follows the initiator, the remaining group members typically follow as well and consensus is reached^12,13^. In this case the initiator is “pulling” other group members into the proposed direction^14^. Collective decisions by consensus can thus be conceived as a special case of individual decision making where each individual’s decision is affected by the decisions of their group mates^15^.

A core question in studies of collective movement is whether specific individuals exert disproportionate influence on group movement. Historically, such individuals have been termed “leaders”^16^. Instances of leadership have been identified both at the start of travel, when individuals attempt to initiate group departures^12,17–21^, and while in motion, when individuals can occupy positions of increased influence, such as the front^7,8,22–25^, or rear^26,27^ of group progressions. A group may follow different individuals over the course of travel as the initiating individual is overtaken by others who occupy the forward, outside positions, or otherwise influence movement from hidden positions within the group^16^. As an example, in their groups, elephant (*Loxodonta africana*) matriarchs set the direction of travel, then are overtaken, and progress at the rear of the group^26^.

Various forms of leadership have been identified, depending on the number and consistency of influential individuals in a group. Some species are characterized by consistent leadership^11^, where the same individual in most cases determines the timing and direction of group movements (e.g., dwarf mongoose, *Helogale parvula*^28^). In other species, leadership is distributed^11^, and the individual determining travel time and direction changes over successive movements (stable leadership e.g. red-fronted lemurs, *Eulemur rufifrons*^29^) or within the same movement (unstable leadership^29^). When leadership is distributed throughout a group, influence over decisions can be shared equally between all group members^30^. Alternatively, leadership can be shared partially among a certain subset of group members that initiate collective movements more often, with greater success, or are more often found at the front of group progressions^11,31^.

The tendency for one or another subset of group members to influence collective movements decisions can be affected by individual characteristics and different facets of the group’s social system^31,32^. Leaders often belong to a certain sex or age category, such as in elephants and killer whales (*Orca orcanus*) where older female group members act as reserves of ecological knowledge and impact group decisions overproportionally^7,26^. The motivational state of individuals, based on their need to acquire vital resources, can additionally effect which individuals initiate departures or are found at the front of progressions^9,10,33,34^. In societies where power differentials play an important role, rank within a dominance hierarchy can be tied to leadership during collective movement. High rank can be associated with more frequently initiating group departures, or traveling at the front of progressions as in the despotic hierarchical society of rhesus macaques (*Macaca mulatta*)^13^. High rank may also be tied to reduced influence during collective movement decisions as in cichlid fish (*Astatotilapia burtoni*) and vulturine guineafowl (*Acryllium vulturinum*)^33,35^. When power differentials are absent, leadership is often more evenly distributed, as in the tolerant, egalitarian society of Tonkean macaques (*M. tonkeana*), where individuals are equally likely to follow any group member, and group progressions exhibit no particular order^13^.

Social organization is also expected to impact coordination of group movement since cohesive uni-level societies are more likely to reach consensus and move as a whole compared to multi-level societies. Multi-level societies are characterized by stable nuclear social units nested within larger predictable aggregations with at least two discernible levels^36^. With multiple social levels, initial movement decisions may originate in core sub-units that further decide to assort according to societal level or move independently^36–38^. Such hierarchical decision processes are of particular relevance in multi-level societies that show certain fission–fusion dynamics. Thus, travel in a multi-level society may be seen as the product of numerous movement decisions made at the level of the core unit, which can lead to upper-level cohesion, or core-/intermediate-level autonomy^10,39,40^. One might then expect the distribution of leadership within multi-level societies to exhibit a particular pattern.

Baboons (genus *Papio*) are an interesting model to study the impact of social factors on leadership during collective movement, as they exhibit considerable variation in their social systems^41,42^. Baboons have been described as having two broad forms of social system, uni-level and multi-level^41^. The “COKY” baboons, chacma (*P. ursinus*), olive (*P. anubis*), Kinda (*P. kindae*), and yellow baboons (*P. cynocephalus*) usually live in uni-level, multimale-multifemale groups^43^, with substantial variation in the steepness of their dominance hierarchies^44^. Studies of group coordination in uni-level baboon societies have shown heterogeneous results^11,34–45^ (Table 1). In some populations, dominant males were more likely to initiate group movements^18,27,57^, while in another population, behaviours associated with initiating collective movement were most conspicuous in older females^46^. There are also reports that adult males initiated more often than females, but both sexes were equally likely to succeed when initiating group departures^12^. Unfortunately, direct comparisons are frequently hampered by differences in methodology (experimental vs. observational, see Table 1)^58^.

**Table 1:** Features of leadership during collective movement in the genus *Papio*. n.a. information not available.

Species	Leadership measurement	Decision context	Study type	Males / Females initiating departures	Decisive factors in reaching departure consensus	Males /Females at front of progressions	High dominance rank linked to leadership
*P. ursinus*	Initiating departures^48^; ^12^; ^74^]	Morning departure from sleeping site^12^; ^74^	Anecdotal observation^48^; ^18^; ^70^; ^53^; ^47^; ^56^	Males^48^; ^12^; ^56^	Troop-mobilizing males^48^	Males^18^; ^70^; ^53^; ^73^	Males^74^; ^71^; ^73^
	Progression order^18^; ^70^; ^53^	Travel throughout the day^48^;^74^ ; ^18^; ^70^; ^53^; ^71^; ^47^; ^56^	Systematic observation^12^; ^74^				
	GPS location^71^		Experimental^73^	Females^12^; ^74^; ^56^	Initiator centrality^74^		No^12^; ^18^; ^47^
	General troop movement^47^; ^73^		Network analysis^74^; ^71^				
			GPS tracking^71^				
*P. cynocephalus*	Pre-departure orienting^46^	Travel throughout the day^46^; ^61^; ^49^; ^69^; ^52^; ^50^	Anecdotal observation^46^; ^61^; ^49^; ^69^; ^52^; ^50^	Males^46^	Decisive males^46^	Males^49^; ^52^	Males^69^; ^52^
	Progression order^61^; ^49^; ^69^; ^52^; ^50^						
				Females^46^	Decisive females^46^	Females^49^; ^52^	Females^46^
						No^61^	No^61^; ^49^
*P. anubis*	Progression order^59^; ^51^; ^54^; ^45^; ^60^	Travel throughout the day^59^; ^51^; ^54^; ^14^; ^31^; ^45^; ^60^	Anecdotal observation^59^; ^51^; ^54^; ^45^; ^60^	Males^14^; ^45^	Decisive females^54^	Males^59^; ^51^; ^54^; ^60^	Males^59^; ^60^
	Initiating departures^14^						
	GPS location^14^; ^31^		GPS tracking^14^; ^31^	Females^54^; ^14^; ^45^	Critical follower number^14^	Females^59^; ^54^	No^14^
*P. kindae*	n.a	n.a	n.a	n.a	n.a	n.a	n.a
*P. hamadryas*	Initiating departures^27^; ^37^; ^57^	Morning departure from sleeping cliff^27^; ^37^; ^57^	Anecdotal observation^27^; ^37^; ^57^	Males^27^; ^37^; ^57^	Decisive males^27^; ^37^; ^57^	Males^27^; ^37^; ^57^	Males^27^; ^37^; ^57^
					Decisive females^57^; ^55^		
					Critical follower number^57^		
*P. papio*	Initiating departures [this paper]	Travel throughout the day [this paper]	Systematic observation [this paper]	Males [this paper]	n.a	Males [this paper]	n.a
	Progression order [this paper]						
				Females [this paper]		Females [this paper]	

Similarly, the likelihood of any particular age or sex category to be found at the front of group progressions is not consistent across species and populations; some groups progress in a male-led order^54^, some with males at the group’s centre^59^, and others with random progression orders^60,61^. Despite wide variation within and between populations and species, it appears that across uni-level baboon species, adult members of both sexes are consistently found at the front of collective movements (Table 1).

Hamadryas (*P. hamadryas*) and Guinea baboons (*P. papio*) live in nested multi-level societies, the base of which are stable reproductive “units” (also: “one-male-units” or OMUs) comprising a single reproductively active adult male (primary male), a small number of adult females and their offspring^27,38,40,62–65^. In hamadryas and Guinea baboon societies males can be differentiated according to their reproductive status, either reproductively active primary males or non-reproductive bachelor males. Bachelor males may be associated with one or more units^27,40,66^. Two to five units and associated bachelor males typically forage and socialize together, forming the second level of the society (“party” in Guinea baboons, “clan” in hamadryas baboons), which in turn come together to form larger groupings (“gangs” or “bands”).

In the multi-level hamadryas baboons, the reproductively active males of the one-male units almost exclusively initiated group movements, often involving complex “negotiations”, in particular before leaving the sleeping sites, while females had only a little impact on group coordination, most likely because female movement was usually hindered by male herding, i.e. keeping females from moving more than a certain distance away^27,40,57^. When on the move, males and sub-adult males appeared at the front of progressions twice as often as would be expected by chance, and were found at the rear of the progression with a frequency equal to chance^27^. In summary, in hamadryas baboons leadership during collective movement appears to be male dominated, mainly driven by the reproductive males of one-male units (Table 1), that were also called “leader males” by Kummer ^27^.

Guinea baboons live in a similar multi-level social organization to hamadryas baboons ^27,38,57^, but their social relationships are characterized by greater male-male tolerance and a higher degree of “female freedom”^62^. If multi-level social organization dictates the distribution of leadership during collective movement, we would expect a similar pattern to emerge as the one reported for hamadryas baboons. However, increased tolerance between males could mean that despite differentiated male reproductive status, all males are equally likely to influence collective movement decisions. In this case, we would expect that both primary and bachelor males can successfully initiate group movements and be equally likely to move at the front of group progressions. In addition, the fact that females have greater leverage in Guinea baboons than in hamadryas baboons, could translate into shared leadership during collective movement. In this case, we would expect that females as well as males can successfully initiate group movements and are just as likely to be found at the front of group progressions as females in uni-level baboon societies.

In all baboon societies, individuals appear to preferentially follow closely affiliated group members regardless of which individual initiates a collective movement^27,40,67^. Olive baboons follow close associates at the start of travel^67^ and hamadryas baboons follow other members of their one-male units and clans^27,37^. Therefore, we expect Guinea baboons will also follow their closest social partners (i.e. members of their units) during departures, and throughout collective movements.

## Results

### Group departures

We sampled a total of 146 attempted group departures, out of which 121 were successful (Table 2). Of the total attempts, 91 (62.3%) were led by adult males [55 by primary males (37.7% of all cases) and 36 by bachelor males (24.7%)], 52 (35.6%) by adult females, and three (2.1%) by juveniles. In two events, the group split as a result of two successful initiation attempts occurring during group departure. Of the 121 successful group departure events, 33 involved only one complete unit, 48 events involved more than one complete unit, and 40 events involved a complete party. Although the sex ratio across the two gangs was nearly 1:1 with 41 adult males and 42 adult females, males were almost twice as likely to initiate group departures than females (see below for statistics). Attempts to initiate group departures came from 58 different individuals: 28 adult males, 27 adult females, and three juveniles. The individuals that attempted initiations most frequently were two primary males, with 11 and seven attempts respectively; followed by four primary males and one bachelor male who each attempted to initiate a group departure six times. The two females that attempted initiations most frequently did so five and four times each.

**Table 2:** Number of attempted and successful initiations collected per study party.

Party	“4”	“5”	“6”	“9”	“10”
Attempted initiations	2	62	34	45	3
Successful initiations	2	50	30	36	3
Progressions	7	47	27	37	6
Hours observed	11.2	261.7	314.7	372.2	10.1

The number of progressions that involved part or all of each study party. The number of hours observer D.M. spent with each party in 2016 and 2017. The main study parties were “5”, “6”, and “9”.

Overall, the predictors age and sex had a clear impact on the probability of attempting an initiation of a group departure (likelihood ratio test comparing full and null model: χ^2^ = 71.882, df = 6, P < 0.001). Being male, primary or bachelor, and of adult age strongly increased the likelihood of attempting an initiation (Table 3).

**Table 3:** Effects of age and sex/reproductive status, as well as unit size, and time of day on the likelihood of attempting to initiate a group departure.

	Estimate	SE	CI_lower_	CI_upper_	χ^2^	P
Intercept	− 1.637	0.379	− 2.38	− 0.894	^(1)^	^(1)^
Sex/reproductive status: Bachelor	0.198	0.347	− 0.048	0.878	14.82	0.569
Sex/reproductive status: Female	− 1.005	0.240	− 1.476	− 0.535	^(2)^	< 0.001
Age: Young	− 3.614	0.720	− 5.026	− 2.201	64.293	< 0.001
Unit size	0.064	0.097	− 0.126	0.253	0.138	0.508
z.time	0.104	0.101	− 0.094	0.301	1.067	0.304
I(z.time^2)	− 0.056	0.063	− 0.180	0.068	0.855	0.376

Reference category is primary male for sex/reproductive status, and adult for age. Estimated coefficients, standard errors, confidence intervals, and test statistics.

^(1)^ not meaningful in this context; ^(2)^ equal values because they refer to different terms of the same variable.

Out of the 52 initiation attempts by adult females, 42 (80.8%) were successful, while out of the 91 attempts by adult males, 79 (86.8%) were successful. Primary males were successful 46 times out of 55 attempts (83.6%), while bachelor males were successful 33 times out of 36 attempts (91.7%). Out of the three initiation attempts by juveniles, two (66.7%) were successful. Once failed, an individual that attempted to initiate tried again only twice in 23 occurrences of unsuccessful attempts. Because only three group departures were initiated by young subjects, we excluded these from further analyses to avoid convergence issues in the statistical models. Neither sex nor male reproductive status explained the variation in individual initiation success (likelihood ratio test: χ^2^ = 0.365, df = 2, P = 0.634, Table S1). In other words, we found no evidence that males, primary or bachelor, were more successful than females at initiating group departures.

When leaving the pre-departure area, subjects that belonged to the same unit were more likely to start moving together. The time intervals between two individuals that belonged to the same unit were significantly shorter (mean = 13.7 s; range: 0—260 s) than the time interval between two individuals who did not belong to the same unit (mean = 25.6 s; range: 0—910 s); likelihood ratio test: χ^2^ = 23.1, df = 1, P < 0.001; Table S2; N = 813 intervals between 104 individuals from 40 events).

### Group progressions

We collected data on 100 events of group progression. Seventeen events involved more than one party. Eleven events involved portions of a party due to a party split lasting most or all of a day. In six of these events, the progressing group consisted of only two units. In order to achieve a comparable number of events per party, the analysis was limited to parties 5, 6, and 9, which were each present in ≥ 27 progression events (Table 2). We excluded parties 4 and 10 which were each present in ≤ 7 events (Table 2).

**Figure 1:**
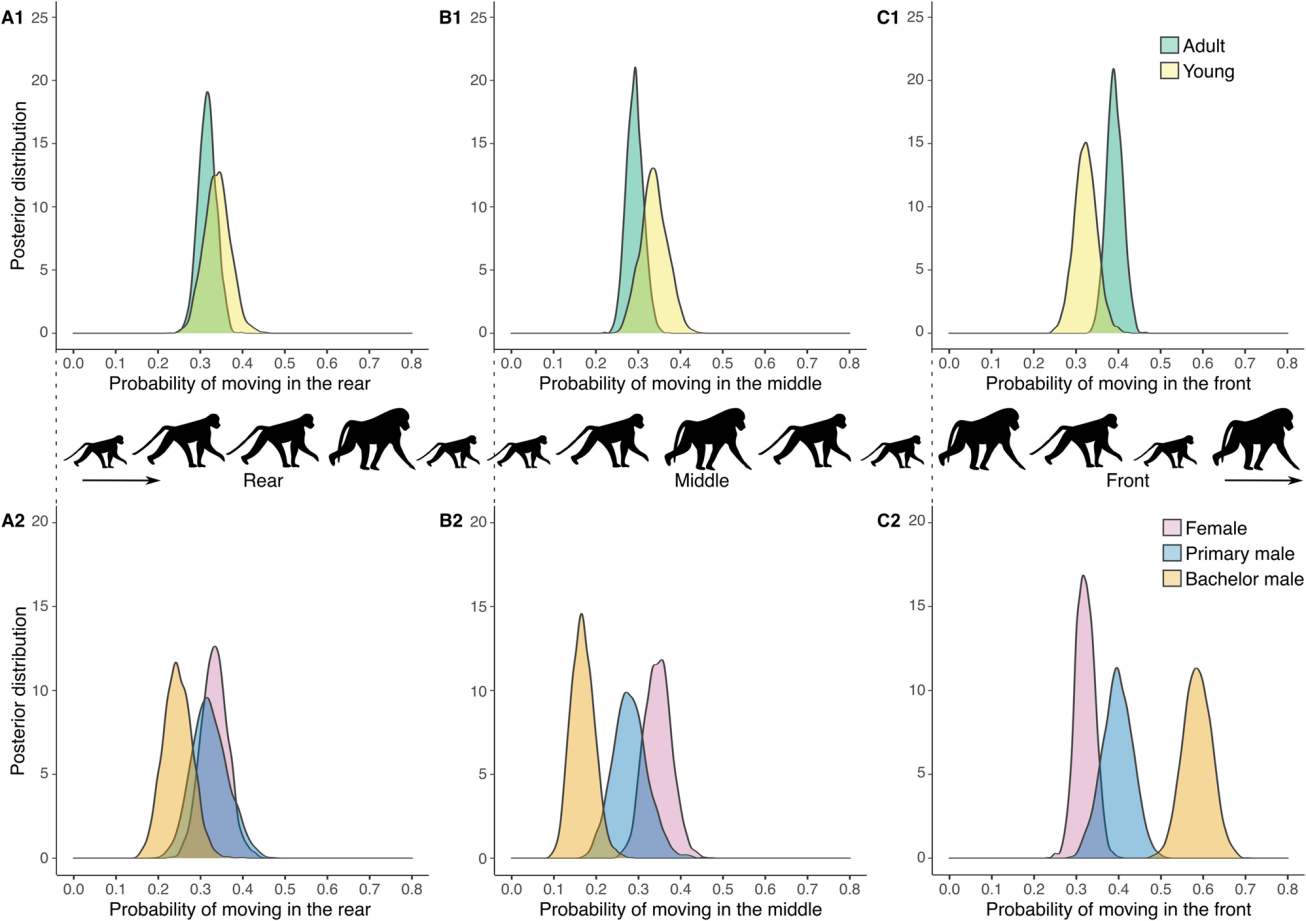
Posterior probability distributions to travel in the (A) rear, (B) middle or (C) front third of a group progression according to (1) age and (2) sex/reproductive status (adult subjects only). The distribution of relative frequency per category per third, i.e. estimated probabilities, in ESM. This figure was made using R (version 3.5.0, https://www.R-project.org)^84^.

Overall, the model outcomes revealed that age partially explained an individual’s position during group progressions (i.e. 95% posterior density intervals do not include 0; Table 4). Adults were more likely to be located in the front third of group progressions than in middle or rear thirds. Young individuals were found in all thirds with a similar likelihood (Fig. 1; the distribution of relative frequencies in Table S3).

**Table 4:** Effect of age (adult, young) on the likelihood for an individual to take a front, middle, or rear position during a group progression.

	Posterior mean	CI_lower_	CI_upper_	Effective sample size	P MCMC
Middle and adult	− 0.294	− 0.503	− 0.113	714.7	0.005
Rear and adult	− 0.213	− 0.403	− 0.001	519.2	0.032
Middle and young	0.339	− 0.005	0.665	688.1	0.049
Rear and young	0.264	− 0.048	0.645	395.5	0.121

Reference categories are the front third of adult and young. Posterior means, confidence intervals, sample size, and P-values derived from MCMC procedure.

We then considered only adult individuals for testing the effect of being a female, a primary male, or a bachelor male on an individual’s position during group progressions. Sex and the distinction between primary and bachelor males explained some variability in the ordering of group progressions (Table 5). Females were found in all thirds with similar likelihood. Primary males were slightly less likely to be found in the middle of the group compared to the front or rear. The strongest effect was observed for bachelor males, that were significantly more likely to move in the front third of the group compared to the middle or rear thirds. The likelihood of observing bachelor males in the front portion of the group differed substantially from that for primary males and females (minimal overlap of distributions; Fig. 1; the distribution of relative frequencies in Table S4).

**Table 5:** Effect of being a female, a primary male, or a bachelor male on the likelihood for an individual to take front, middle or rear positions during a group progression.

	Posterior mean	CI_lower_	CI_upper_	Effective sample size	P MCMC
Middle and female	0.0742	− 0.195	0.332	813.8	0.582
Rear and female	0.037	− 0.229	0.299	607.0	0.784
Middle and primary male	− 0.427	− 0.911	0.022	596.3	0.074
Rear and primary male	− 0.250	− 0.700	0.218	726.6	0.306
Middle and bachelor male	− 1.334	− 1.788	− 0.861	604.4	< 0.001
Rear and bachelor male	− 0.912	− 1.346	− 0.482	580.4	< 0.001

Reference categories are the front third or each sex/reproductive status category. Posterior means, confidence intervals, sample size, and P-values derived from MCMC procedure.

During group progressions, subjects that belonged to the same unit were more likely to travel together, as evidenced by the interval time between two individuals belonging to the same unit (mean = 4.2 s; range: 1—70 s), which was significantly shorter than the interval time between two individuals that did not belong to the same unit (mean = 8.9 s; range: 1—293 s; likelihood ratio test: χ^2^ = 201.5, df = 1, P < 0.001; Table S5; N = 2226 intervals between 120 individuals following in 100 events).

## Discussion

We provide evidence that the Guinea baboons in our study population coordinate collective movements through partially shared consensus (distributed leadership), where most adult group members can successfully initiate group departures and move at the front of group movements^68^. Adult males attempted initiations more often than adult females, but members of both sexes were highly successful when attempting to initiate group departures. Primary and bachelor males attempted initiations with similar frequency and they were similarly successful. During group progressions, bachelor males were more likely to be found at the front, while primary males and females were found in all portion with similar probability. Individuals of the same unit typically departed and travelled together. Thus, sex, male reproductive status (primary or bachelor), and unit membership affected group departure and group movement patterns differentially.

## Baboon leadership and intersexual dynamics

The patterns of individual influence over collective movement decisions that we observed during group departures in Guinea baboons were overall more similar to those seen in uni-level baboon species such as chacma, yellow, and olive baboons^12,14,45,46^ rather than those of hamadryas baboons^27,37^. In Guinea baboons, males initiated group departures more often than females, but both males and females initiated group departures with similar degrees of success. This is precisely the pattern found in a group of chacma baboons^12^ and fits the general trend of partially-shared consensus observed in populations of olive, yellow, and chacma baboons^14,45,46,54^. The order in which Guinea baboons progressed during travel also reflected the shared influence of males and females over collective movement decisions. Female Guinea baboons occupied front, centre, and rear positions with similar likelihood. When in front, females could potentially influence ongoing movement decisions similar to females in some troops of yellow, olive and chacma baboons^12,14,69,70^. In addition, the male tendency to travel at the front was weaker in Guinea baboons compared to hamadryas baboons, where males were twice as likely to walk at the front of progressions than predicted by chance^27^. Positions at the rear of progressions were equally taken by individuals of all age, sex and male reproductive status categories, differing from patterns where males were more frequently observed toward the rear of hamadryas and chacma baboon progressions^27,70^. Thus, multi- or uni-level social organization per se does not directly translate into one or another type of leadership during collective movement.

The tendency of one or both sexes to display leadership during group departures and travel could instead be a reflection of inter-sexual relationship within a society. This could explain the striking similarity between the patterns of leadership in uni-level “COKY” baboons and the multi-level Guinea baboons, as well as the differences between hamadryas and all other baboon societies. Female baboons in uni-level societies exhibit a high degree of social and physical freedom, living as they do in groups centred on a matrilineal dominance hierarchy of varying steepness^43,44^. Females and males socialize and move independently, mainly interacting in and around periods when females are fertile or have young offspring ^43,44^. In contrast, male and female hamadryas baboons remain together year round – a proximity enforced by males herding females^27,40,57^. Although Guinea baboons have stable male–female associations in one-male units like hamadryas baboons, female Guinea baboons are quite independent and spend a substantial amount of their time away from males^62^. If aggressed, female Guinea baboons sometimes respond with counter-aggression and even form female-female coalitions against males^62^, a phenomenon rarely observed in hamadryas baboons^27^. Females also transfer between primary males independently, moving freely between all three levels of the social system to join a primary male^62^. Female Guinea baboons thus exhibit a degree of social freedom similar to females “COKY” baboons^62^. In conclusion, the divide in consensus decision-making between hamadryas and all other baboon species could be the result of differences in the degree of physical and social mobility of males and females, rather than a consequence of their multi-level social organization.

Importantly, the case of hamadryas baboons may not be as cut and dry as it is widely held to be. Both Kummer^27^ and Stolba^57^, who mainly focused on males during their studies of group departures from sleeping rocks, reported anecdotes where females affected the departure process by failing to follow a departing male, thus thwarting his initiation attempt^27,57^. In such cases, hamadryas females acted as an anchor, similar to individual olive baboons that did not follow an initiation, leading the initiator to return to the group^14^. Adult male hamadryas baboons initiating group departures were successful on average only 60% of the time, meaning 40% of attempted departures did not garner followers. The “amoeba-like” morning departure process, with its many false starts involving only a portion of the group, could be a result of hidden female hamadryas influence over collective movement decisions, and not only the effect of “vetos” from other males during the negotiation process.

### Traveling as a unit

Analysis of time intervals between traveling individuals revealed that members of the same unit were more likely to depart and travel together in close proximity than members of different units. This finding fits a trend seen in other baboon species. Both uni-level and multi-level baboons preferentially follow closely bonded group members regardless of who initiates a movement^27,71–74^.

Individuals assorting by unit while on the move could explain why classes of individuals that make up Guinea baboons units, i.e. primary males, females, and young, were found in all portions of progressions with similar frequencies. In contrast, bachelor males were more likely to be found in the front of the progression than in its middle. The tendency of bachelor males to be at the front of group progressions could indicate that, once on the move, they are choosing the direction of group movement. Yet, we found no evidence that bachelor males initiated departures more often or more successfully than primary males. Alternatively, bachelor males may be more likely to move at the front of progressions simply because they travel faster than their unit-bound party members (see Harel, Loftus, and Crofoot 2020^75^).

### Examples from other multi-level species

There is no clear pattern of leadership in collective movement across multi-level societies. The more core the level of social organization, the more time members of the social sub-unit spend together in all contexts—including travel. Fission of upper levels occurs during travel for some species ^7,76,77^, but a persistent finding in multi-level societies is that core units travel together synchronously [plains zebras (*Equus burchelli*)^10^, geladas (*Theropithecus gelada*)^78^, vulturine Guineafowl^33^, hamadryas baboons^27^, sperm whales (*Physeter macrocephalus*)^77^, black and white snub-nosed monkeys (*Rhinopithecus bieti*)^79^]. Which members of a core unit more frequently initiate departures or occupy influential positions within progressions varies between both taxa and population^39^. The same characteristics such as age^7,24^, dominance rank^33^, motivation^10^, and sex^27^ that affect the distribution of leadership in uni-level societies also affect multi-level societies. Thus, multi-level societies do not necessarily have a characteristic means of reaching collective movement decisions, instead nested social levels are simply one variable contributing to the challenge of group coordination.

## Conclusion

Overall, the results presented here demonstrate that the uni-level or multi-level organization of a society alone does not determine how baboon groups reach consensus about time and direction of travel. Despite the similarities of hamadryas and Guinea baboon social organization, in the Guinea baboons we observed a partially-shared consensus style of decision-making more similar to patterns described in some troops of uni-level savannah baboons than to the male dominated style described in hamadryas baboons. We have highlighted how social tolerance between group members could result in a pattern of collective movement leadership where all adults—females, primary males, and bachelor males—can initiate group departures with a high degree of success, and travel at the front of group progressions. Furthermore we discussed how a preference for following close associates could lead Guinea baboons to depart and travel in social sub-units.

Speaking more broadly, studies of collective movement in nonhuman primates should also consider that in a number of species, groups have typical travel routes within their home ranges. Consequently, once an initial travel direction has been chosen, there may in fact be little more to decide^57,72^. Future work could examine the extent to which travel decisions are guided by habitual use of familiar paths compared to a more situational, case-by-case form of decision making.

## Materials and methods

### Field site and study subjects

The fieldwork was based at the field station “Centre de Recherche de Primatologie (CRP) Simenti” (13°01′34″ N, 13°17′41″ W), in the Niokolo-Koba National Park, south-eastern Senegal. The study site lies next to the Gambia River, where multiple seasonal wetlands (Mare) occur in depressions alongside the river and the prevailing vegetation types are dry forests and various savannah types, including savannah woodlands, tree/shrub savannahs, and grass savannahs^80^. The habitat can be defined as comparatively rich in resources for Guinea baboons (more details in Zinner et al. 2021^80^). The multi-level system of Guinea baboons consists of “units” (usually one adult male and one to several females with their young), units are nested within “parties” and parties are nested within “gangs”^38^. The study subjects were fully habituated baboons belonging to five parties that formed two gangs (Table 6). Subjects were individually identified by natural markings, body shape and size, and radio collars. The identification of juveniles was not always possible because of their changing body features. From a previous study we know, the home ranges of the parties covered on average 30.3 km^2^ of largely overlapping territories (Kernel density estimations 95%)^80^.

**Table 6:** Average composition of study parties.

Gang	Party	Number of units	Number of adults		Size
“Mare”	“4”	2–3	5 ♂	3 ♀	15
	“9”	5–6	12 ♂	17 ♀	45
	“10”	1–2	2 ♂	2 ♀	8
“Simenti”	“5”	3–4	10 ♂	9 ♀	25
	“6”	4–5	12 ♂	11 ♀	38

Party sizes (i.e. total number of party members) varied due to births, deaths, disappearances, between-parties transfers of individuals, and difficulties in recognizing young weaned individuals.

### Data collection

We collected all data in 2016 and 2017 from January to August, respectively, 6 days per week. Observation days began before sunrise (at 6:00 or 6:30) in order to locate baboons at their sleeping sites. We recorded data on Samsung Galaxy Note 3 handhelds using forms created with Pendragon 7.2 (Pendragon Software Corporation, USA). D.M. took all data on departure and progression, and together with other team members, he collected census, ad libitum, proximity scan, and focal data of the baboons to investigate demography, reproductive success, association data, and behavioural patterns^81^. Observer reliability was regularly checked. We used focal follows and ad libitum data of grooming, copulations, contact-sitting, and aggressions to validate female-male associations, following the procedure described in Goffe and colleagues (2016)^62^. Group movement data were recorded from all instances of travel that occurred during daily observations (i.e. all-occurrence sampling^81^). We distinguished two types of events during the group movement process: group departures and group progressions.

### Operational definition of group departures

We collected data on events of group departures throughout the day, whenever suitable conditions arose. Here we define “group” as an assemblage of animals comprising one or more complete units or a complete party. Once the group was stationary (the position of the group did not change for at least 15 min) we began checking whether any individuals attempted to initiate a group departure (comparable to e.g., ^8,29,68,82,83^). An individual was defined as initiating a group departure if it moved outside a set pre-departure area, defined as a circle of maximum 20 m diameter that encompassed the group. The edge of a pre-departure area had to be at least 20 m away from other baboons, to avoid potential influences from baboons not considered in the departure event on a focal group’s movement decisions. In Guinea baboons, 20 m proved to be a useful measure of spatial association because males of the same party frequently remain within 20 m of one another, while males of different parties are rarely found within 20 m ^63^. The identity of all individuals moving away from the pre-departure area, their leaving time, and their direction of movement were voice recorded. The first individual leaving the pre-departure area was labelled an initiator, and any individuals moving away from the pre-departure area in the same direction as, and within 5 min of, the initiator were considered followers. Following established methods, an individual leaving the pre-departure area at an angle > 45° to left or right of the direction taken by an initiator and/or leaving > 5 min after an initiator was coded as an initiator of a separate departure attempt^13,29^. An initiation was considered successful if all or part of the group in the pre-departure area followed. When two successful initiations were coded in one event, this was labelled group fission. We excluded movements prompted by predation risks, alarm calls, or social interactions such as threats or chases.

### Operational definition of group progressions

Group progression was defined as a group of baboons traveling in approximately single-file in largely the same direction. Single-file travel typically occurs along pathways such as dirt tracks and open grassy areas. We collected group progression data whenever a group consisted of one or more complete units and when it had been at least 30 min since the last recorded group progression. Progression data were collected after the researcher visualized a reference line on the ground in front of the advancing group. For each baboon that crossed the reference line, we recorded its identity (or age-sex category) and time of crossing to the nearest second, using a handheld voice recorder.

### Data analyses

For the analysis, we categorized individuals according to age, sex, and male reproductive status. Individuals in the “young” category included infants, yearlings, and small and large juveniles; individuals in the “adult” category included all subadults and adults. We categorized individuals according to sex. Males were further separated by reproductive status into primary males (males associated with females in a unit) and bachelor males (males associated with no females or single unit). We further noted the unit an individual belonged to for primary males, females, and identifiable juveniles. In Guinea baboons, bachelor males may associate with multiple units ^66^, thus bachelor males were not assigned membership to any particular unit. The same rule was applied for juveniles that could not be clearly identified. In addition, we considered unit size in the analysis, defined as the number of adult subjects in the unit. All models and plots were fitted in R (version 3.5.0; R Core Team, 2018)^84^, using RStudio interface (version 1.1.383; RStudio Team, 2016).

### Group departures

To test whether the likelihood of attempting to initiate a group departure was predicted by sex/reproductive status (primary male, bachelor male, and female), age (young vs. adult), and unit size, we fit a Generalized Linear Mixed Model (GLMM^85^) with a binomial response variable and logit link function. Sex/reproductive status, age, and unit size were included in the model as fixed effects. Individual identity and event number were included as random effects to control for variation related to individuals or single movement events. A polynomial function of the time of day was included as an additional predictor variable (standardized to avoid scaling issues), to control for any variation in movement pattern related to differences between morning and afternoon travel. We used the glmer function in the lme4 R package (version 1.1–17^86^), setting the optimizer to ‘bobyqa’ to prevent convergence issues. To test if the global model fit better than a simpler alternative we compared the full model to a null model containing only the random effects and time with a likelihood ratio test^87^. P-values of individual effects we obtained by dropping them from the full model, one at a time, and comparing the respective reduced models with the full model. All model comparisons were based on likelihood ratio tests using the drop1 function in the lme4 R package (argument ‘test’ set to ‘Chisq’; version 1.1–17^86^). Confidence intervals for regression coefficients were obtained by bootstrap using the bootMer function provided in the package lme4 (nboots = 1000). In a second step, using the same procedure, we tested whether sex/reproductive status, age, and unit size predicted whether or not an individual’s initiation attempt was successful.

To approximate distances between individuals and investigate the spatial association of individuals within parties, we calculated the interval times between successive individuals to the nearest second. We restricted the analysis of interval times to 40 events where at least one complete party was present, and calculated interval times only for individuals that could be identified, thereby excluding most juveniles. To test whether interval times were influenced by unit identity, we used a linear mixed model (LMM; Baayen 2008) in which we included unit identity as a fixed effect and included the identity of the following individual and event number as random effects. The model was fitted using the lmer function of the lme4 R package (version 1.1–17^86^). The interval times were highly skewed and therefore log-transformed before analysis. We verified that the assumptions of normally distributed and homogeneous residuals were met by visually inspecting a qqplot and a plot of the residuals against the fitted values. We tested model stability by excluding subjects one by one from the dataset and comparing the estimated models obtained from these subsets with the one obtained on the full dataset. This procedure revealed no influential subjects. We tested whether the full model fit the data significantly better than a null model in which the fixed effect was omitted, using the ANOVA R function (argument test ‘Chisq’^87,88^). The models were fitted using the Maximum Likelihood option^89^. The p-value results from a likelihood ratio test comparing the full with the reduced model, using the drop1 function (argument ‘test’ set to ‘Chisq’^90^).

### Group progressions

To test whether age or sex/reproductive status predicted the distribution of individuals during a group progression we used a multinomial logit regression model with random intercepts ^91^. We divided the sequence of individuals within a progression into equal thirds and coded progression-location into three categories (front, middle, and rear) with the probability of being found in a given third dependent on age and sex/reproductive status category. The model was estimated using a Bayesian method. We obtained posterior densities of the regression coefficients with the Markov-chain Monte Carlo (MCMC) method, using the MCMCglmm R package^92^. We used the resulting regression coefficients of progression-location from the posterior samples to calculate the probability p (i.e. the distribution of the relative frequency) to observe individuals of a given age (young vs. adult) or sex/reproductive status (female, primary male, bachelor male) in the front, middle or rear of a group progression ESM formula set 1 & 2).

Finally, we investigated the spatial association within the group progression to test whether interval times were influenced by unit membership, as with group departures. Using the time interval between successive individuals within a progression, we applied the same procedure as was used for the analysis of interval times in group departures. In brief, we used a linear mixed model (LMM^85^) that included unit membership as a fixed effect, and the identity of the following individual and event number as random effects.

### Ethics declaration

This study was non-invasive and strictly observational. ﻿Research was conducted within the regulations set by Senegalese agencies as well as by the Animal Care Committee at the German Primate Center.

### Data availability

The dataset and code used in the current study are available at https://osf.io/dg3hz/?view_only=62ee196d7d3040c4b261ede03fdc4ead.

## Supplementary Information

Supplementary Tables.
