# Supplementary material for: Coordination during group departures and progressions in the tolerant multi-level society of wild Guinea baboons (Papio papio)
Source: Sci Rep. 2021 Nov 9;11:21938. doi: 10.1038/s41598-021-01356-6 (PMC8578668; doi:10.1038/s41598-021-01356-6)
Supplement: Supplementary file 1 — Supplementary Tables. [file 41598_2021_1356_MOESM1_ESM.pdf]

Electronic Supplemental Materials for: Coordination during group departures and progressions in the tolerant multi-level society of wild Guinea baboons (Papio papio)

Davide Montanari, William J. O'Hearn, Julien Hambuckers, Julia Fischer & Dietmar Zinner

Table S1. Effects of age (adult, young) and sex/reproductive status (primary male, bachelor male, female), as well as unit size, and time of day on the likelihood of successfully initiating a group departure. Estimated coefficients, standard errors, confidence intervals, and test statistics.

	Estimate	Std. Error	CI_{lower}	CI_{upper}	χ^2	P
Intercept	2.03323	1.06705	-0.058	4.12	(1)	(1)
sex/reproductive status: Bachelor	0.46843	0.98386	-1.460	2.397	0.365	0.6340
sex/ reproductive status: Female	-0.14485	0.53625	-1.196	0.906	(2)	0.7871
unit size	-0.13940	0.25105	-0.631	0.353	0.306	0.5787
z.time	-0.05438	0.27133	-0.586	0.477	0.040	0.8411
I(z.time^2)	0.20069	0.28039	-0.348	0.750	0.672	0.4742

Table S2. Summary of linear mixed model results. Coefficient estimates of the effect of belonging to the same unit, on the time interval between two individuals joining a group departure event.

	Estimate	Std. Error	CI_{lower}	CI_{upper}	Df	t value	Pr(>|t|)
(Intercept)	2.303	0.076	2.150	2.458	50.475	29.947	< 2e-16
Belonging to the same unit	-0.394	0.078	-0.549	-0.238	554.001	-5.016	7.11e-07

Table S3. Estimated probabilities to travel in the rear, middle, or front third of a group progression according to age (adult, young). Values are calculated from the posterior mean values of the model.

	Rear	Middle	Front
Adult	0.316	0.292	0.391
Young	0.339	0.337	0.322

Table S4. Estimated probabilities to travel in the rear, middle, or front third of a group progression according to sex/reproductive status (female, primary male, bachelor male). Values are calculated from the posterior mean values of the model.

	Rear	Middle	Front
Female	0.333	0.346	0.321
Primary male	0.322	0.281	0.397
Bachelor male	0.246	0.168	0.585

Table S5. Summary of linear mixed model results. Coefficient estimates of the effect of belonging to the same unit, on the time interval between two individuals traveling in a group progression event.

	Estimate	Std. Error	CI_{lower}	CI_{upper}	Df	t value	Pr(>|t|)
(Intercept)	1.598	0.044	1.51	1.685	124.66	36.2	<2e-16
Belonging to the same unit	-0.500	0.032	-0.565	-0.436	817.282	-15.24	<2e-16
